# Bacteriome dataset from the rhizosphere of trees in a *Pinus pinaster* and *Pinus halepensis* dominated forest subjected to drought conditions

**DOI:** 10.1016/j.dib.2022.108805

**Published:** 2022-12-05

**Authors:** Ana V. Lasa, M. Ángeles Guevara, Pablo J. Villadas, Antonio J. Fernández-González, María Teresa Cervera, Manuel Fernández-López

**Affiliations:** a Department of Soil and Plant Microbiology, Estación Experimental del Zaidín, CSIC, Profesor Albareda 1, 18008, Granada, Spain; b Department of Forest Ecology and Genetics, Instituto de Ciencias Forestales, ICIFOR-CN-INIA-CSIC, Carretera de La Coruña km 7,5, 28040, Madrid, Spain; c Mixed Unit of Forest Genomics and Ecophysiology, INIA-UPM, Madrid, Spain

**Keywords:** Rhizosphere bacteriome, 16S rRNA gene, metabarcoding, Mediterranean pine forest, Microbial ecology, Drought

## Abstract

The Mediterranean basin is drastically affected by intense and frequent droughts, which jeopardize the diversity and survival of its forest, for example, *Pinus pinaster* forests. The dynamics of the bacterial communities inhabiting the rhizosphere of *Pinus pinaster* and other plants from a pine dominated forest under contrasting hydric conditions was monitored. The forest was located in Sierra de Oria (southeast Spain), and it was mainly composed by *P. pinaster, P. halepensis*, woody shrub species and herbaceous plants. 18 trees visually belonging to *P. pinaster* located along the perimeter and across the forest were selected for the analysis. All the trees were separated at least 50 m each other. Although all of them belonged to *P. pinaster* morphologically according to visual identification, the genotyping of the roots confirmed that they corresponded to *P. pinaster, P. halepensis*, and other plant species different from genus *Pinus*, although in the last case it was not possible to identify the plant species. At a distance less than 50 cm from the trunk, the litter and topsoil were removed, and the soil closely attached to non-suberified roots (rhizosphere soil) was collected (depth of 5-25 cm). Sampling was carried out in two seasons with contrasting temperature and rainfall patterns: on July 18, 2017 (summer) and April 24, 2018 (spring). After rhizosphere soil DNA and RNA extraction (and cDNA synthesis), a metabarcoding approach was followed by sequencing the V3-V4 hypervariable regions of the *16S rRNA* gene and its derived transcripts by Illumina MiSeq platform. Sequencing reads were bioinformatically processed; specifically, they were filtered, trimmed, clustered into ASV (Amplicon Sequence Variants), and taxonomically identified. As a result, a total of 1,123,209 and 1,089,359 quality sequences were obtained from DNA and RNA-derived libraries, which resulted in 5,241 and 5,231 ASVs, respectively. Total communities (DNA) were mainly dominated by phyla *Proteobacteria, Acidobacteria, Actinobacteria, Verrucomicrobia* and *Bacteroidetes* in summer and spring, while potentially active populations (RNA libraries) were rich in *Proteobacteria, Acidobacteria, Candidate division WPS-1, Actinobacteria* and *Verrucomicrobia* both in summer and spring. On the other hand, DNA libraries were mainly dominated by genera *Sphingomonas* and acidobacterial groups Gp4 and Gp6, while potentially active bacteria (RNA) were rich in acidobacterial Gp3, Gp4, Gp6 and *Phenylobacterium*, although their relative abundance depended on the considered season.

This dataset can provide valuable information about bacterial candidates which could be used as bioindicators of drought conditions. In addition to shifts in the bacterial relative abundance due to seasonal changes, the ratio RNA-based cDNA:DNA could be calculated as proxy of the potential activity of bacterial taxa. Moreover, these data could aid in developing bioformulations based on microorganisms which could be resistant to desiccation and involved in the drought resistance mechanisms of the host plant.

**Specifications Table**

**Table:** 

Subject	Biological sciences
Specific subject area	Microbiology: Microbiome
Type of data	Table Figure Fastq files
How the data were acquired	Rhizosphere RNA and DNA extraction was performed by means of RNA PowerSoil® Total RNA Isolation Kit and the RNA PowerSoil® DNA Elution Accesory kit (MoBio). The retrotrascription of RNA to cDNA was achieved by molecular methods. The Illumina MiSeq platform was employed for the sequencing of V3-V4 hypervariable regions of the gene *16S rRNA* by 2 × 300 pb strategy (PE 300) and three replicates of a mock community were employed as quality control of the sequencing (ZymoBIOMICS Microbial Community Standard II in log distribution). Bioinformatic processing of the raw reads was performed with DADA2 package of R software, and downstream data processing was also performed with R.
Data format	Raw Analyzed
Description of data collection	18 rhizosphere soil samples were collected from trees belonging to *Pinus pinaster, P. halepensis* and other plant species from a forest located in Sierra de Oria (Almeria, Spain) in summer and spring. DNA and RNA were extracted from samples coming from both seasons, obtaining a total of 72 samples. RNA was retrotranscribed to cDNA, and each sample was sequenced independently. Samples coming from one tree were discarded due to the low number of quality sequences retained.
Data source location	Region: Sierra de Oria Province: Almería Country: Spain Latitude and longitude for collected samples: 37⁰ 31´N 2⁰ 21´W
Data accessibility	Data are available at the National Center for Biotechnology Information Sequence Read Archive (NCBI SRA) repository under the BioProject number PRJNA748008. https://www.ncbi.nlm.nih.gov/bioproject/?term=PRJNA748008
Related research article	A.V. Lasa, M.A. Guevara, P.J. Villadas, M.D. Vélez, A.J. Fernández-González, N. de María, M. López-Hinojosa, L. Díaz, M.T. Cervera, M. Fernández-López. Correlating the above- and belowground genotype of *Pinus pinaster* trees and rhizosphere bacterial communities under drought conditions. Sci. Total. Environ. 832 (2022) 155007. http://dx.doi.org/10.1016/j.scitotenv.2022.155007

## Value of the Data

• The dataset provides information about the composition of bacterial communities inhabiting the rhizosphere of *Pinus pinaster, P. halepensis* and other plant species located in a pine forest subjected to severe droughts, both during the summer and spring, at DNA and RNA levels (total and potentially active bacteria, respectively). The data can help to gain more insights into the dynamics of total and potentially active bacteria under contrasting water conditions.
• The data could be useful in comparative analyses of total and potentially active bacteria, bacterial communities collected in spring and summer seasons, populations inhabiting different plant species in a pine dominated forest, even to compare with bacterial populations inhabiting the rhizosphere of plants located in other forests. The effect of rainfall levels on rhizosphere bacterial communities could also be studied.
• These data can be valuable for other microbiologists interested on the dynamics of rhizosphere bacterial communities, for researches focused on forest microbial ecology, even for forestry agents interested on gaining more insights into the status of Mediterranean forests. This dataset could also be used by stakeholders to implement conservation and sustainable forest management programs.
• The data could be used in other studies to analyze the effect of the climate change on bacterial communities, to find biomarkers of extreme drought events, to seek for bacterial candidates potentially resistant to water deficits, or to develop bioformulations based on drought tolerant bacteria.
• This dataset could be useful for further studies aimed at conserving Mediterranean forests in a scenario of climate change, and could be applied in other parts worldwide characterized by Mediterranean climate.

## 1 Objective

Mediterranean ecosystems are a great source of biodiversity, although they are threatened by climate change. The increasing temperatures and the drastic changes in rainfall regimes jeopardize the stability and even the survival of Mediterranean biomes, especially forest ecosystems. The forest under study is located in Sierra de Oria (Almeria, Spain), dominated mainly by trees belonging to *Pinus pinaster* (which exhibit remarkable adaptability to drought) and *P. halepensis* species. This site is representative of dry Mediterranean mountain areas, strongly subjected to the effects of climate change. The adaptive response of *P. pinaster* to drought could be partly mediated by its associated microbiota, since changes in plant hosts usually result in a selection and recruitment of certain microorganisms, triggering microbial metabolic processes involved in drought tolerance [1]. Thus, we sequenced the V3-V4 hypervariable regions of the gene *16S rRNA* from the DNA and RNA extracted from the rhizosphere of different plants (mainly *P. pinaster, P. halepensis* trees and other plant species) in summer and spring in order to gain more insights into the seasonal dynamics of rhizosphere prokaryotic communities. Other researchers interested in the effects of climatic change over microbial communities or working in the same experimental area could leverage this dataset, adding value to the original research article already published [2].

## 2 Data Description

The dataset deposited in the SRA of NCBI included 75 files in fastq format, including the raw reads of Illumina sequencing of the V3-V4 hypervariable regions of the gene *16S rRNA*. 36 files corresponded to the DNA samples, among which 18 were related to the samples collected in summer (2017) and the other 18 in spring (2018). In the case of the cDNA obtained from the rhizosphere RNA, 18 samples corresponded to those collected in summer and the remaining 18, in the spring. The dataset also included 3 replicates of a mock community including the DNA of an artificial microbial community composed by taxonomically identified microorganisms. It should be noted that the number of reads of the RNA-derived samples corresponding to the tree 14 (summer) was markedly low (< 100 reads), so all the samples collected from that tree were removed from the dataset.

Considering the remaining 17 trees, a total of 2,018,364 and 2,081,876 raw sequences were obtained in the DNA and RNA-derived libraries, respectively. After the trimming and filtering steps, 1,123,609 (DNA library) and 1,089,359 (RNA-derived library) high-quality sequences were retained, which resulted in a total of 5,241 and 5,231 total and potentially active ASVs. Table 1 shows the number of raw and high-quality sequences obtained in each season and library.

**Table 1:** Summary of the high-throughput sequencing output.

Library	Season	Raw sequences	High-quality sequences
**DNA**	Summer	977,744	534,720
	Spring	1,040,620	588,489

**RNA**	Summer	1,015,691	514,497
	Spring	1,066,185	574,862

**Fig. 1:**
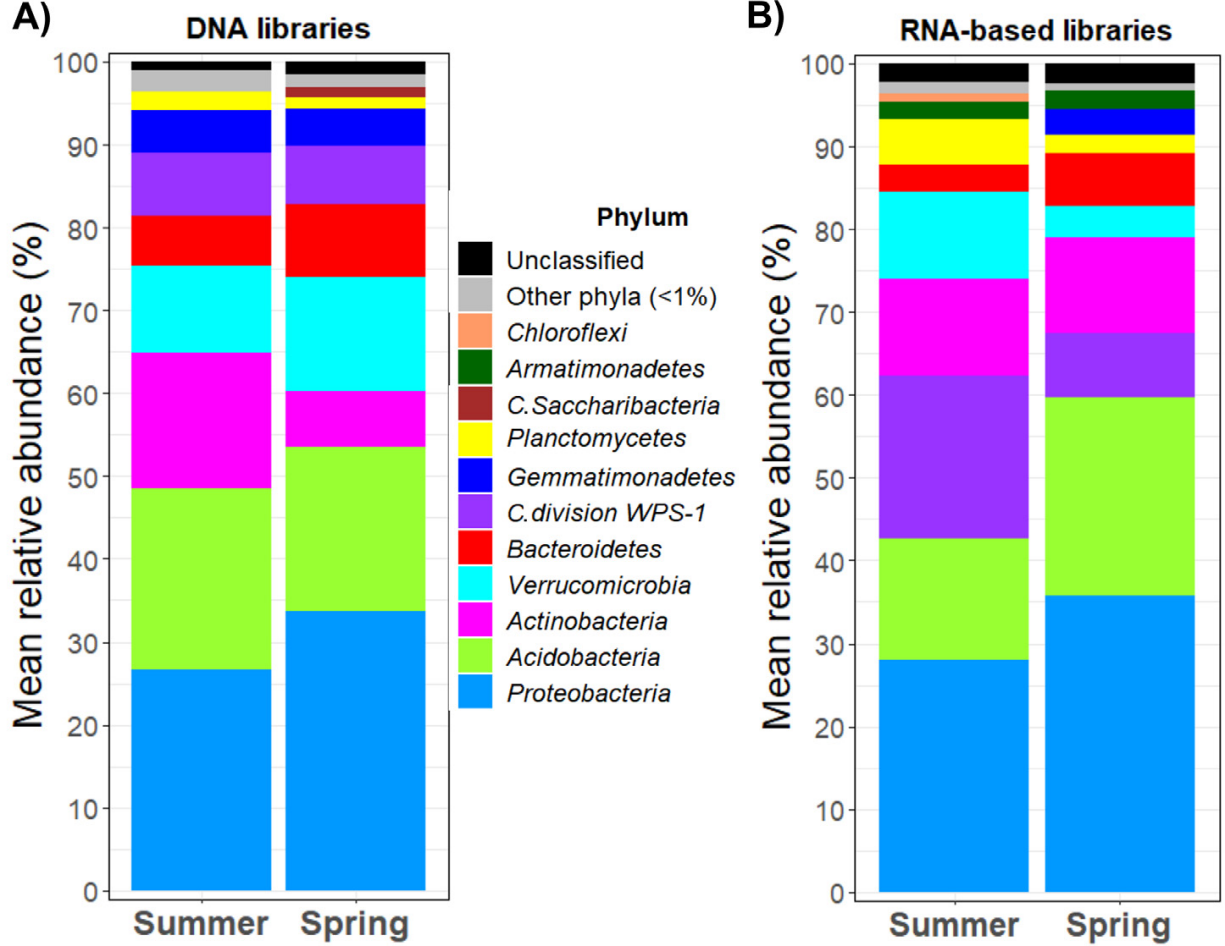
Mean relative abundance of the total (A) and potentially active (B) bacterial phyla dwelling in the rhizosphere of *P. pinaster, P. halepensis* or other plants in the pine forest located in Sierra de Oria, in spring and summer. All the phyla that accounted for < 1% of the total sequences were clustered together in the artificial group “Other phyla”. “C.” refers to *Candidate*.

No archaeal sequences were detected in the whole dataset, and the mean relative abundance of those sequences that could not be identified with any already known bacterial phyla was below 2.4% (Fig. 1). In the DNA library, sequences belonging to 18 and 19 bacterial phyla were detected in the case of samples collected in spring and summer, respectively. Notwithstanding, sequences ascribed to phyla *Proteobacteria, Acidobacteria, Actinobacteria, Verrucomicrobia* and *Bacteroidetes* accounted more than 80% of the total sequences in both seasons (Fig. 1A). Potentially active bacteria were mainly dominated by phyla *Proteobacteria, Acidobacteria, Candidate division WPS-1, Actinobacteria* and *Verrucomicrobia,* accounting these phyla for more than 80% of the total sequences included in the RNA-derived libraries in both seasons (Fig. 1B). Going into deeper taxonomical levels, total bacterial communities (DNA) were composed of 276 classified genera in spring, while 258 were detected in summer. It should be noted that more than 37% of the total sequences could not be ascribed to any known bacterial genera included in the taxonomical database. Among classified genera, the mean relative abundance of acidobacterial Gp4 and Gp6 and *Sphingomonas* was considerably high, accounting for more than 15.9 and 18.5% of the total sequences registered in spring and summer, respectively (DNA; Table 2). On the other hand, 48 and 34.8% of the RNA-derived sequences recorded in summer and spring, respectively, were not identified with any already classified genera. Nevertheless, 272 classified genera were detected in summer, while 277 were registered in spring. Potentially active bacterial communities were mainly dominated by acidobacterial Gp3, Gp4, Gp6, *Phenylobacterium* and *Sphingomonas* in summer, while spring was characterized by bacterial communities rich specially in acidobacterial Gp3, Gp6 and *Phenylobacterium* (Table 2).

**Table 2:** 10 most abundant rhizosphere bacterial genera in DNA and RNA-derived libraries, in both seasons. Data indicate the mean relative abundance, considering the abundance of each taxon in the 17 trees analyzed.

DNA						
Phylum	Class	Order	Family	Genus	Summer	Spring
*Proteobacteria*	*Alphaproteobacteria*	*Sphingomonadales*	*Sphingomonadaceae*	*Sphingomonas*	5.35 ± 1.79	6.99 ± 2.18
*Acidobacteria*	*Acidobacteria Gp4*	*Acidobacteria Gp4 ois*	*Acidobacteria Gp4 fis*	*Gp4*	7.72 ± 5.64	3.83 ± 2.89
*Acidobacteria*	*Acidobacteria Gp6*	*Acidobacteria Gp6 ois*	*Acidobacteria Gp6 fis*	*Gp6*	5.38 ± 2.07	5.06 ± 2.67
*Proteobacteria*	*Alphaproteobacteria*	*Rhizobiales*	*Bradyrhizobiaceae*	*Bradyrhizobium*	2.78 ± 1.26	2.49 ± 0.82
*Actinobacteria*	*Actinobacteria*	*Mycobacteriales*	*Mycobacteriaceae*	*Mycobacterium*	2.73 ± 2.35	1.71 ± 2.15
*Acidobacteria*	*Acidobacteria Gp3*	*Acidobacteria Gp3 ois*	*Acidobacteria Gp3 fis*	*Gp3*	1.60 ± 0.89	2.34 ± 0.70
*Proteobacteria*	*Alphaproteobacteria*	*Rhodospirillales*	*Reyranellaceae*	*Reyranella*	1.07 ± 0.42	1.41 ± 0.65
*Acidobacteria*	*Acidobacteria Gp16*	*Acidobacteria Gp16 ois*	*Acidobacteria Gp16 fis*	*Gp16*	1.83 ± 1.18	0.49 ± 0.37
*Bacteroidetes*	*Sphingobacteriia*	*Sphingobacteriales*	*Sphingobacteriaceae*	*Mucilaginibacter*	0.49 ± 0.68	1.78 ± 1.59
*Proteobacteria*	*Alphaproteobacteria*	*Micropepsales*	*Micropepsaceae*	*Micropepsis*	1.03 ± 0.47	1.22 ± 0.56

RNA						
*Acidobacteria*	*Acidobacteria Gp3*	*Acidobacteria Gp3 ois*	*Acidobacteria Gp3 fis*	*Gp3*	2.58 ± 1.66	7.05 ± 2.05
*Acidobacteria*	*Acidobacteria Gp6*	*Acidobacteria Gp6 ois*	*Acidobacteria Gp6 fis*	*Gp6*	2.80 ± 1.53	5.25 ± 2.88
*Acidobacteria*	*Acidobacteria Gp4*	*Acidobacteria Gp4 ois*	*Acidobacteria Gp4 fis*	*Gp4*	4.30 ± 4.42	2.63 ± 2.42
*Proteobacteria*	*Alphaproteobacteria*	*Caulobacterales*	*Caulobacteraceae*	*Phenylobacterium*	2.87 ± 1.58	2.99 ± 1.26
*Proteobacteria*	*Alphaproteobacteria*	*Sphingomonadales*	*Sphingomonadaceae*	*Sphingomonas*	2.67 ± 1.74	2.70 ± 1.26
*Armatimonadetes*	*Chthonomonadetes*	*Chthonomonadales*	*Chthonomonadaceae*	*Chthonomonas/Armatimonadetes gp3*	1.55 ± 1.10	1.53 ± 1.05
*Actinobacteria*	*Thermoleophilia*	*Solirubrobacterales*	*Solirubrobacteraceae*	*Solirubrobacter*	1.40 ± 1.01	1.55 ± 0.99
*Proteobacteria*	*Alphaproteobacteria*	*Rhizobiales*	*Bradyrhizobiaceae*	*Bradyrhizobium*	2.14 ± 1.19	0.53 ± 0.24
*Acidobacteria*	*Acidobacteria Gp1*	*Acidobacteria Gp1 ois*	*Acidobacteria Gp1 fis*	*Gp1*	0.24 ± 0.70	2.35 ± 2.75
*Proteobacteria*	*Deltaproteobacteria*	*Myxococcales*	*Kofleriaceae*	*Kofleria*	1.28 ± 0.93	1.29 ± 1.09

*ois,* order *incertae sedis*

*fis,* family *incertae sedis*

## 3 Experimental Design, Materials and Methods

### 3.1 Sample collection

A forest dominated by *Pinus pinaster* and *P. halepensis* trees (subjected to severe droughts) was selected in Sierra de Oria (Almeria, Spain; 37⁰ 31’ N 2⁰ 21’ W). 18 *P. pinaster* trees (visually identified), separated at least 50 m from each other, were selected and their needles genotyped as described by Lasa and collaborators [2]. A summary of the specific sampling points is given by Lasa and colleagues [2]. Rhizosphere soil samples were taken in summer (July 18, 2017) and spring (April 24, 2018), when trees were subjected to severe water stress and reduce their vegetative growth (mean temperature and precipitation in June and July 2017: 23.9°C and 1.6 mm), and in the wet season (mean temperature and precipitation in March and April 2018: 12.1°C and 26.5 mm), when the vegetative growth is intense, respectively. At a distance of less than 50 cm from the trunk, litter and topsoil were removed and the main roots of each tree were followed by digging until young roots were found (active roots). For each tree, roots were harvested and genotyped, identifying samples from *P. pinaster* trees as well as samples from *P. halepensis* or other plant species. 2 g of the soil closely attached to the non-suberified roots was taken by rubbing them at a depth of 5-25 cm. Each rhizosphere soil sample was then mixed with 5 mL of the LifeGuard™ Soil Preservation Solution (MoBio Laboratories), needed for stabilization of microbial RNA in soils. Samples were stored at 4 ⁰C until they were processed in the laboratory within 24 h of sampling.

### 3.2 Rhizosphere DNA and RNA extraction and cDNA synthesis

Total RNA and DNA from each individual rhizosphere soil sample were extracted by means of the RNA PowerSoil® Total RNA Isolation Kit and the RNA PowerSoil® DNA Elution Accesory kit (MoBio), respectively. Double stranded cDNA was synthesized as described previously [3]. Specifically, soil RNA was treated with DNase I (Roche) and RNase Out Recombinant Ribonuclase Inhibitor (Invitrogen) to remove the contaminant DNA. The absence of DNA was confirmed by trying to amplify the gene *16S rRNA* by PCR [4]. Since no amplification was achieved, single stranded cDNA was synthesized using SuperScript^TM^ II Reverse Transcriptase (Invitrogen) and random primers. A treatment with RNase R (Roche), DNA polymerase I (Promega) and DNA ligase (Invitrogen) was performed in order to synthesize double stranded cDNA. Finally, blunt-end cDNA was created using T4 DNA polymerase (Invitrogen). The quantity of extracted DNA and synthesized cDNA was measured by fluorometry using Qubit 3.0 (Life Technologies).

### 3.3 High-throughput sequencing

The V3-V4 hypervariable regions of the prokaryotic gene *16S rRNA* were sequenced through the Illumina MiSeq platform at the Genomics Unit of the Institute of Parasitology and Biomedicine López-Neyra (IPBLN-CSIC, Granada, Spain). This institution was the responsible of sequencing library preparation. For this purpose, DNA and cDNA were used as template, and Pro341F and Pro 805R [5] as forward and reverse primers, respectively. Amplicons were sequenced by following a paired-end 2 × 300 bp (PE 300) strategy. It should be noted that three replicates of an artificial mock microbial community (ZymoBIOMICS Microbial Community Standard II in logarithmic scale, ZYMO RESEARCH) were included in the same sequencing run as positive control. The mock community helped in stablishing the detection limits of the sequencing, due to the already known taxonomic composition of the artificial microbial community.

### 3.4 Sequencing data processing

Raw reads were processed bioinformatically by means of DADA2 package of R [6], following the developers’ tutorial (https://benjjneb.github.io/dada2/tutorial.html). Data processing included a first step of quality filtering and trimming in which low quality sequences obtained with forward and reverse primers were discarded from the dataset. Reads with ambiguities and more than two expected errors were also removed (function *filterAndTrim* of the DADA2 package). As suggested the DADA2 pipeline, parametric error of the model was calculated and corrected from the data (functions *learnErrors* and *dada*). Then, the forward and reverse sequences were overlapped by using the default parameters (function *mergePairs*). An Amplicon Sequence Variant (ASV) table was generated by means of the function *makeSequenceTable*, and those sequences of a different length than that corresponding to the sequenced hypervariable regions of the gene *16S rRNA* were removed from the dataset (other different to 401-429 bp). Subsequently, a chimera detection and removing step was performed by the function *removeBimeraDenovo* included in the package DADA2.

Quality sequences were classified taxonomically by comparing them to those included in the reference database of the Ribosomal Database Project (RDP-II) training set v.18 (function *assignTaxonomy*). Finally, the ASVs that showed a relative abundance lower than the detection limit stablished with the mock community were removed from the dataset.

## Ethics Statements

Pine material was obtained according to the national ABS legislation before the Nagoya Protocol on Access to Genetic Resources, and the Fair and Equitable Sharing of Benefits Arising from their Utilization to the Convention on Biological Diversity was legally implemented by signatory countries.

## CRediT Author Statement

**Ana V. Lasa:** Conceptualization, Methodology, Software, Formal analysis, Writing-Original Draft. **M. Ángeles Guevara**: Conceptualization, Writing-Review. **Pablo J. Villadas:** Resources. **Antonio J. Fernández-González:** Methodology. **María Teresa Cervera:** Conceptualization, Resources, Writing-Review & Editing, Supervision, Funding acquisition. **Manuel Fernández-López:** Conceptualization, Methodology, Resources, Writing-Review & Editing, Supervision, Project administration, Funding acquisition.

## Declaration of Competing Interest

The authors declare that they have no known competing financial interests or personal relationships that could have appeared to influence the work reported in this paper.

## Data Availability

Microbiome dataset obtained by Illumina Miseq sequencing (Original Data) (NCBI SRA).
